# Surgical site infection and antimicrobial prophylaxis prescribing profile, and its determinants among hospitalized patients in Northeast Ethiopia: a hospital based cross-sectional study

**DOI:** 10.1038/s41598-023-41834-7

**Published:** 2023-09-06

**Authors:** Habtemariam Alekaw Habteweld, Mohammed Yimam, Abate Wondesen Tsige, Yehualashet Teshome Wondmkun, Bedilu Linger Endalifer, Kassahun Dires Ayenew

**Affiliations:** Department of Pharmacy, Debre Berhan University, P.O. Box 445, Debre Berhan, Ethiopia

**Keywords:** Microbiology, Diseases, Health care, Risk factors

## Abstract

The inappropriate use of surgical antimicrobial prophylaxis is a common cause for increased risk of morbidity and mortality from surgical site infection in patients who underwent surgical procedures. The study aimed to evaluate surgical antimicrobial prophylaxis prescribing patterns, Surgical Site Infection (SSI), and its determinants in the surgical ward of Debre Berhan Comprehensive Specialized Hospital, northeast Ethiopia. A prospective cross-sectional study was conducted from October 1st 2022 to January 31st, 2023. Data collected from patient medical record cards and patient interviews were entered and analyzed using SPSS V26.0. The determinants of surgical site infection were determined from the multivariable logistic regression. P-value ≤ 0.05 was considered statistically significant. Ceftriaxone (70.5%) followed by a combination of ceftriaxone with metronidazole (21.90%) was the most frequently used prophylactic antibiotic. One hundred fifty-nine (78%) of patients were exposed to inappropriately used prophylactic antimicrobials and 62.2% of these were exposed to inappropriately selected antibiotics. One hundred twenty-six (61.9%) patients developed Surgical Site Infection (SSI). Duration of procedure longer than an hour and inappropriate use of antimicrobial prophylaxiswere the independent predictors for the occurrence of surgical site infections. Patients whose operation was lasted in longer than an hour were 3.39 times more likely to develop SSI compared to those whose operation was completed in less than an hour, AOR = 3.39 (95% CI: 1.24–9.30). Similarly, controlling the effect of other covariate variables, individuals who were given inappropriate antimicrobial prophylaxis were 6.67 times more likely to develop SSI compared to those given appropriate prophylaxis, AOR = 6.67 (95% CI: 1.05–42.49). The high rate of SSI requires due attention from clinicians as well as health policymakers. Duration of surgical procedure greater than an hour and inappropriate antimicrobial prophylaxis use was the independent predictor of surgical site infections.

## Introduction

Patients may develop Surgical Site Infections (SSI) at or near surgically incised sites within 30 days of their operation or after 1 year if the procedure underwent have an implant which was placed^1,2^. Several factors like wound classification, the type of surgical procedure, the skills of the surgeon, the duration of surgery, antimicrobial prophylaxis appropriateness, and patient-related factors, such as preexisting comorbidities (poorly controlled diabetes mellitus, extreme of age, cigarette smoking, alcohol abuse, and immune compromising conditions) can affect the occurrence of SSI^3,4^. Surgical Antimicrobial Prophylaxis (SAP) plays an important role in reducing SSI, especially if patient-related risk factors for SSIs are present^3,5^. When appropriately used, surgical antimicrobial prophylaxis is an effective method for preventing the occurrence of SSI^6,7^. For optimal benefit, determining the appropriate indication based on definitive microbiological tests, and timing, selecting an agent that covers the likely pathogen on wound contamination, and administering sufficient bactericidal concentrations during the whole period that the incision is open for risk of bacterial contamination is required^6–8^.

Surgical site infection is one of the most common nosocomial infections causing complications of operative procedures which have a significant burden in terms of morbidity, mortality, and healthcare costs. SSI is the third among all reported cases of inpatient nosocomial infections. Accounted for up to 16% of nosocomial infections in all hospitalized patients and 38% of all surgical patients^2,9^. Based on the wound's level of contamination, there are four classes of surgical wounds: clean (wound with no inflammation), clean-contaminated (wound in which the respiratory, alimentary or genitourinary tracts are entered but without contamination), contaminated (wound where acute inflammation (without pus) is encountered, or where there is visible contamination of the wound.), and dirty-infected (wound in the presence of pus, where there is a previously perforated hollow viscous or compound/open injury more than four hours old)^9,10^.

Development of SSIs results in up to 60% increased risk of spending time in an intensive care unit, being readmitted to a hospital, and nearly doubled mortality rates compared to patients without SSIs^1^. World Health Organization (WHO) estimated that the burden of nosocomial infection from SSI in low- and middle-income countries ranges from 1.2–23.6% and this incident is three to five times higher than in high-income countries due to the poor infection prevention programs, crowding hospital environment and irrational prescription of antimicrobial agents^2,8,11,12^. Irrational use of prophylactic antibiotics is associated with increased medical care costs, prolonged hospitalization, superinfection, the emergence of poly antimicrobial-resistant strains of hospital pathogens that challenge the patient care process, and adverse drug reactions, and this phenomenon is being aggravated by the absence of culture and sensitivity tests in treating infections^2^. Although the data is limited, in Ethiopia, 9.4–75% of patients who underwent surgical procedures developed SSI at different hospitals^13–16^. Furthermore, a report from a meta-analysis study indicated that the estimated pooled occurrence of SSI in Ethiopia was 12.3%^11^. From an estimated 30–50% of the antimicrobials used for surgical prophylaxis in hospitals, 30–90% were inappropriate^17^. The use of surgical antimicrobial prophylaxis before any surgery in an appropriate way is an important measure to prevent SSI. However inappropriate use of antimicrobial agents like inappropriate selection, timing, and duration are associated with an increase in the worldwide emergence of antimicrobial resistance, a major public health problem, and has a significant impact on treatment and outcomes. Moreover, there will be also increased risks of occurrence of surgical site infections, and adverse drug reactions thus strongly calling for the evaluation of SAP^15,17^.

There are no studies conducted on surgical antimicrobial prophylaxis and surgical site infections at Debre Berhan Comprehensive Specialized Hospital (DBCSH). Therefore, this study aimed to assess SSI and surgical antimicrobial prophylaxis utilization patterns, appropriateness, and associated factors among patients admitted to the surgical ward of DBCSH. Investigating the appropriateness of surgical prophylactic antibiotics use and subsequent occurrence of SSI at the ward level can generate detailed information about the magnitude and composition of antibiotic consumption and give insight into the rationality of prescribing in the process of patient care. This will help policymakers to make recommendations, monitor, evaluate, and suggest modifications in the level of practice and diagnostic techniques, and practitioner’s prescription habits, so as to make patient care reasonable and effective. The generated data and recommendations will be utilized also in the future for the promotion of rational antibiotic use in DBCSH.

This study will also be used as a pilot study for national surveillance studies regarding antibiotic utilization and resistance which will change the unregulated use of antibiotics for common diseases. Furthermore, the findings of this study can be used as a baseline for further studies conducted in this area.

## Methods

### Study area and study period

This study was conducted at DBCSH which is located in Debre Berhan town in Amhara National Regional State, Ethiopia, at a distance of 130 Kilometers from Addis Ababa, and 695 Kilometers from Bahir Dar the regional capital. DBCSH is a governmental hospital in the town serving the population of the surrounding area as a referral center. The Hospital has four major wards including Medical, Surgical, Pediatrics, and Gynecology and Obstetrics. The hospital has 4 surgeons and the surgical ward unit is equipped with around 39 beds and serves an average of 80 people a month. The study was conducted from October 1st, 2022 to January 31st, 2023.

### Study design

A 4-month facility-based prospective cross-sectional study design was used to assess surgical antimicrobial prophylaxis prescribing pattern, Surgical Site Infection (SSI), and its determinants in the surgical ward of DBCSH, Northeast Ethiopia.

### Source population

All patients admitted to the surgical ward of DBCSH for surgical procedure.

### Study population

All patients who were admitted to the surgical ward of DBCSH from 1st October 2022 to 31st January 2023 for surgical procedures during the study period and those who fulfilled the inclusion criteria.

### Eligibility criteria

#### Inclusion criteria

Adult patients of age > 18 years, patients on at least one prophylaxis antibiotic, and wounds classified as clean, clean-contaminated, and contaminated are considered as inclusion criteria.

#### Exclusion criteria

Patients who received therapeutic antimicrobials during hospitalization before surgery for a comorbid condition and those with a history of previous admission for a similar surgical procedure were excluded from the study. Patients with incomplete information were also excluded from the study.

### Sample size determination and sampling technique

All patients admitted to the surgical ward of DBCSH for the surgical procedure during the study period that fulfilled the inclusion criteria were included. A total of 357 patients were admitted during the four-month study period in the surgical ward, 86 patients were excluded as they had already dirty wounds, 47 took antibiotics prior to surgery (within 48 h) for other reasons, 4 patients were excluded as they have repeated admission for surgical cases similar to their previously done surgery and 16 were excluded due to incomplete information. Finally, a total of 204 patients were found to be eligible for this study and were taken as the final sample size.

### Variables of the study

Surgical site infection occurrence is the dependent variable, Patient-related factors such as (age, sex, residence), and surgical procedure-related factors such as duration of surgery, Presence of concomitant drugs, Amount of bleeding, co-morbidity, history of previous surgery wound class, and appropriateness of prophylaxis, are the independent variables.

### Data collection procedure

The data collection tools for this study were adapted based on American Society of Health-System Pharmacists (ASHP) guidelines on SAP for surgery and different literature reviewed^7,8,11^. The data extraction form was utilized to collect data on patient characteristics, diagnosis, types of surgical procedure, and SAP received including indication, choice, duration, time of the first dose, and other perceived factors associated with SSI. Patient characteristics of socio-demographic information were taken directly through interviewing the patients. The complete chart review was done for all patients until discharge. ASHP guideline, UpToDate version 21.6, and specific guidelines from the Ministry of Health of Ethiopia were used for evaluating the appropriateness of SAP used. Prophylactic surgical antimicrobials were registered as appropriate when the right antimicrobial is utilized to the right indication at the right time, and for the right duration during the surgical procedure as per the above references.

### Data processing and analysis

The collected data were entered, coded, and analyzed using SPSS version 26.0 software. Tables and figures with frequencies and percentages were used to present the results of this study. The bivariate and multivariable logistic regression analysis models were used to find out the determinants of the occurrence of surgical site infection. Variables that have a significant association at p-value < 0.25 in the bi-variate analysis were taken to multi-variate logistic regression analysis to include all potential variables. The odds ratio and their 95% CI were used to indicate the strength of association between independent and outcome variables. P-values of less than 0.05 were considered statistically significant.

### Data quality assurance

The data collection format was pre-tested on 15 individuals and necessary adjustment to the data collection tool was made accordingly. The collected data were checked daily for consistency, correctness, and completeness. When data failed to be complete it was discarded and alternative data were taken from the subsequent medical records.

### Ethical consideration

Before any attempt to collect data, ethical clearance was obtained from the ethical review board of Debre Berhan University, Asrat Woldeyes Health Sciences Campus, and an official permission letter was obtained from the general director of Debre Berhan Comprehensive Specialized Hospital. Informed consent for the study was obtained from each study participant and/or their legal guardian(s) before data collection and documented in a prepared format. Privacy of the data was also assured and collected anonymously. Moreover, all methods in the present study were performed in accordance with the declarations of Helsinki.

### Operational definitions

Surgical Antimicrobial Prophylaxis (SAP): Refers to the administration of antimicrobial agents prior to the beginning of the operation.

Surgical site infection: is an infection occurring at or near a surgical incision within 30 days of operation or after one year in case of an implant and affecting either the incision or the deep tissue in parts of the body where the surgery took place. This is only confirmed by clinical signs and symptoms of surgical site infections.

Antimicrobial prophylaxis outcome: is a term used to indicate the presence or absence of surgical site infections.

Appropriate Surgical Antibiotics Prophylaxis: using the right antimicrobial when only indicated at the right time and for the right duration during the surgical procedures.

Right antimicrobial: Antimicrobials that are prescribed inexpensive, non-toxic, and limited-spectrum and are in accordance with local and national policies and guidelines^6,7^.

Right duration: Prescribing the shortest antibiotic course (not more than 24 h) that is likely to be effective for the presenting patient’s condition. Be discontinued within 24 h after the end of surgery, to prevent the emergence of resistance.

Appropriate timing of surgical antimicrobial prophylaxis: defined as the administration of antimicrobials in a range of 30 to 60 min prior to the incision to achieve prophylactic level during surgery and optimize efficacy as per the predetermined ability of antimicrobials to be administered to reach peak levels in tissue.

## Result

### Socio-demographic related factor

In this study, a total of 204 patients were assessed for surgical antibiotic prophylaxis use, appropriateness, and outcome. As depicted in Table 1, nearly three-fourths (73.5%) of patients were males. The minimum age of the study participants was 18 years while the maxim was 77 years old and their mean age was 34.13 ± 12.8. About 54.3% of the study participants were residents of rural areas.

**Table 1:** The sociodemographic and clinical characteristics of patients admitted to the surgical ward of DBCSH from October 1st, 2022 to January 31st, 2023.

Characteristic		Frequency	Percent (%)
Sex	Male	150	73.5
	Female	54	26.5
Age in years	18–40	154	75.5
	> 40	50	24.5
Social drug user	Yes	41	20.1
	No	163	79.9
Comorbidity	Yes	45	22.1
	No	159	77.9
Type of comorbidity	Hypertension	18	40
	PUD	10	22.2
	HIV	6	13.3
	Diabetes Mellites	6	13.3
	Others	5	11.1
Types of Surgery	Emergency	157	77
	Elective	47	23
Wound class	Clean	49	24
	Clean contaminated	130	63.7
	Contaminated	25	12.3
Duration of surgery	≤ 1 h	88	43.1
	> 1 h	116	56.9
Types of surgical procedures conducted	Gastrointestinal surgery	146	71.6
	Orthopedic surgery	21	10.3
	Urological surgery	6	2.9
	Hernia repair surgery	19	9.3
	Head and neck surgery	2	1
	Other surgery	10	4.9
Blood transfusion	Yes	25	12.3
	No	179	87.7
Total	204		

Other surgery includes: appendectomy, Graft, and Breast surgery.

*indicate Deep Vein Thrombosis, and Psychosis.

*DBCSH* Debre Berhan Comprehensive Specialized Hospital, *PUD* Peptic Ulcer Disease, *HIV* Human Immunodeficiency Virus.

Regarding the clinical characteristics of patients, only 45 (22.1%) of patients have comorbid conditions, and the most common comorbid condition identified was hypertension 18 (40%) followed by peptic ulcer disease 10 (22.2%). The majority (79.9%) of patients included in the study don’t have a history of regular use of social drugs like alcohol, cigarettes, coffee, and khat. The clean contaminated wound was the most commonly encountered wound class which accounted for 130 (63.7%) of all cases. More than two-thirds, 157 (77.1%) of the patients underwent emergency surgery while the rest were elective surgery. The most common type of surgery encountered in this study was gastrointestinal surgery (146, 71.6%) while the least type was head and neck surgery (2, 1%). Only 25 (12.5%) of patients need a blood transfusion to undergo the surgical procedure (Table 1).

### Utilization pattern and appropriateness of SAP

**Figure 1:**
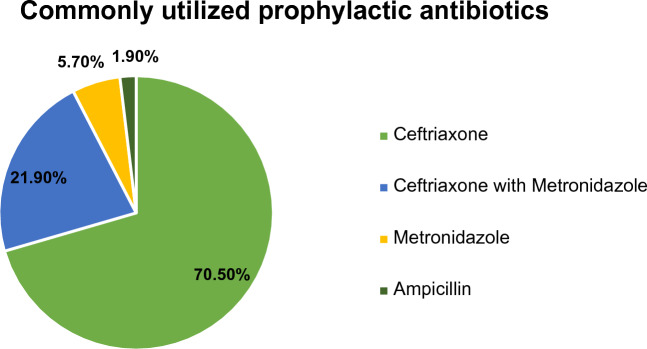
Pattern of Prophylactic Antibiotic Use in patients admitted to the surgical ward of DBCSH from October 1st, 2022 to January 31st, 2023.

The patient included in the current study was given SAP with one or two antibiotics for the prevention of SSI. Ceftriaxone (70.5%) followed by the combination of ceftriaxone with metronidazole (21.90%) was the predominantly used prophylactic antibiotic. On the other hand, ampicillin was the least used prophylactic antibiotic (1.9%) (Fig. 1).

**Figure 2:**
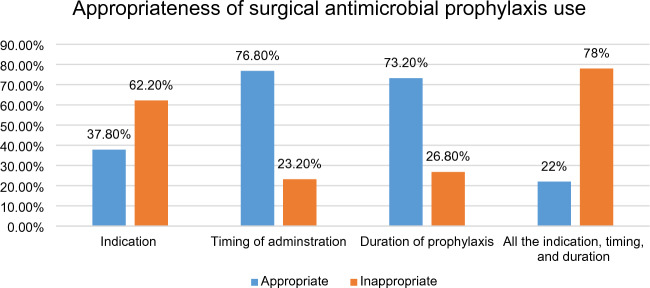
Appropriateness of surgical antibiotic prophylaxis utilization in patients admitted to the surgical ward of DBCSH from October 1st, 2022 to January 31st, 2023.

In this study, 159 (77.9%) patients were exposed to inappropriate use of surgical antimicrobial prophylaxis as per different guidelines^6,7^. This inappropriateness was related at least to inappropriate time of administration, drug selection, duration of prophylaxis, and or a combination of these problems. The highest proportion (62.2%) of patients were exposed to inappropriately selected antibiotics followed by prolonged duration of prophylaxis (26.8%) (Fig. 2).

### Occurrence of SSI

In our study 126 (61.8%) patients have developed SSI, of which 73 (58%) were superficial incisional SSI and the rest were deep incisional SSI. The infection rate was higher among patients with a contaminated (80%) than the clean (30.6%), and clean-contaminated wound (70%) class of wounds. There was also a relatively higher occurrence of SSI in patients with comorbidity (73.3%) than those with no comorbid conditions (58.5%). The majority (93.6%) of patients developed SSI before they were discharged from the hospital (Table 2).

**Table 2:** The occurrence of SSI among admitted patients who underwent a surgical procedure at the surgical ward of DBCSH from DBCSH from October 1st, 2022 to January 31st, 2023.

Variables	Frequency	Percent (%)
Occurrence of SSI		
Present	126	61.8
Absent	78	38.2
Time of occurrence of SSI		
Before discharge	118	93.6
After discharge	8	6.4
Type of SSI		
Superficial incisional	73	58
Deep incisional	53	42

### Determinants of surgical site infection occurrence

There are many factors that determine the occurrence of surgical site infection. In this study, there was a higher rate of surgical site inection in patients where surgical procedure lasts for longer than an hour than those whose procedure was completed in less than an hour, (AOR = 3.39; 95% CI: 1.24–9.30). In addition, surgical site infection was higher in patients who were exposed to inappropriately used surgical antimicrobial prophylaxis (AOR = 6.67; 95% CI: 1.05–42.49) (Table 3).

**Table 3:** Determinants of surgical site infection occurrence among patients admitted to the surgical ward of DBCSH from October 1st, 2022 to January 31st, 2023.

Variables		Surgical site infection		Odds ratio	
		Yes (%)	No (%)		
		126 (61.8)	78 (38.2)	COR (95% CI)	AOR (95% CI)
Presence of concomitant drugs	Yes	109	47	2.99 (1.14–7.87)	2.32 (0.62–8.63)
	No	17	31		
Previous surgery	Yes	23	29	4.30 (0.91–20.35)	4.18 (0.71–24.50)
	No	103	49		
Wound class	Clean	15	34	2.18 (0.86–5.29)	1.07 (0.32–3.53)
	Others	111	44		
Amount of bleeding	≤ 100 ml	69	59	2.57 (1.07–6.11)	1.69 (0.54–5.31)
	> 100 ml	57	19		
Prophylaxis antibiotics used	Appropriate	11	34	9.07 (1.99–41.21)	6.67 (1.05–42.49) *
	Inappropriate	115	44		
Duration of surgery	≤ 1 h	35	53	5.42 (2.30–12.77)	3.39 (1.24–9.30) *
	> 1 h	91	25		
Duration of hospitalization	≤ 5 days	31	39	3.06 (1.32–7.08)	2.12 (0.71–6.33)
	> 5 days	95	39		

*Statistically Significant: P ≤ 0.05.

Others represent a combination of clean-contaminated and contaminated wounds.

*AOR* adjusted odds ratio, *COR* crude odds ratio, *CI* confidence interval.

## Discussion

Determination of surgical antibiotic prophylaxis outcome is essential for the reduction of morbidity; unnecessary hospital stays and related costs by revealing the level of evidence required to avoid inappropriate use of drugs during the management of a patient undergoing surgery with prophylaxis^18^. Hence, this study was carried out to assess surgical antimicrobial prophylaxis outcomes and associated factors among patients admitted to the surgical ward of DBCSH, which was a hospital located in the northeast of Ethiopia.

The current study showed that 61.8% of patients developed SSI. This finding was higher than the finding of previous studies conducted in different Hospitals in Ethiopia that reported 10.2–23.4% of patients developed SSI^1,9,10,13,15,16,18,19^. The higher occurrence of SSI in our study might be due to the lack of adequate infection control systems, and inappropriate use of antibiotics like ceftriaxone in surgical antimicrobial prophylaxis which is not indicated due to its lower activity against bacteria commonly encountered in elective surgery including gram-positive cocci which are the common organisms causing SSIs or it may be due to differences in the type of surgical procedures, professional differences, and operation environmental and patient-related factors including the presence of immune compromising comorbidities like HIV and diabetes. Moreover, it might be also due to the absence of recommended prophylactic antibiotics use due to the lack of a culture and sensitivity study. This means the pathogens responsible for surgical site infections may not have been covered by the prophylactic antibiotics or they may have developed resistance to these agents.

Nonetheless, the prevalence was lower than in a study conducted at Ayder Teaching and Referral Hospital^14^, where a 75% prevalence of SSI is reported. This could be because the previous study has been done on patients who are clinically suspected to be having SSI.

In this study, almost all of the SSIs identified (93.6%) have occurred before discharge from the hospital. This finding is in line with a prospective study conducted at Wachemo University Nigist Eleni Mohammed Memorial Hospital which reported that 90.47% of patients developed SSIs in the hospital^20^. This similarity in the timing of occurrence of SSI could be due to the similarity in the level of care provided to patients during their hospital stay. It might be also due to the high prevalence of undetected pathogens causing an infection in both settings and the absence of an antimicrobial stewardship program that enables the prudent use of antimicrobials.

In this study, the majority of surgical procedures underwent were clean-contaminated surgeries (63.7%) which is in line with a study conducted at Borumeda Hospital where a higher number of patients (48%) have a clean-contaminated wound. However, it opposed the finding of other studies^2,8,11,20^ conducted at different times in different areas where the majority of procedures (38.1%, 40.5%, 54%, and 56.5%) respectively were clean.

Regarding the type of SSI identified in this study, the majority (58%) of them were superficial incisional while the rest 42% were deep incisional infections, which is similar to the finding of other studies^2,15,19^. The highest prevalence of superficial incisional infections might be due to the higher prevalence of pathogens responsible for causing skin and soft tissue infections. However, the finding was not in line with a retrospective review which reported about 54.6% superficial or deep incisional SSI, and 45.4% organ/space SSI^21^. In our study, no organ/space SSI was identified. This variation in the type of SSI might be due to the difference in the type of procedure, quality of care, or variation in the patient-related risk factor.

Selection of antimicrobials for surgical site infections should be based on the antimicrobial coverage against suspected pathogen types causing SSI which may vary depending on anatomical location, operation type, and patterns of local antimicrobial resistance. Moreover, the cost of antimicrobials, pharmacokinetics, and narrowness in the spectrum of activity should be taken into account in their selection^11^. Though different guidelines claimed cefazolin as the first choice of surgical prophylaxis antimicrobial agent^6,7,17^, ceftriaxone was the most commonly used (70.5%) antibiotic for surgical prophylaxis in this study. The use of third-generation cephalosporins like ceftriaxone in surgical antimicrobial prophylaxis is not indicated due to their lower activity against bacteria commonly encountered in elective surgery including gram-positive cocci which are the common organisms causing SSIs, and their higher cost^17^. This finding was in line with a similar study conducted in different parts of Ethiopia^2,8,11,15,17,18,21,23^. The high utilization rate of ceftriaxone in the present study might be due to the unavailability of first and second-generation cephalosporins, the availability of ceftriaxone, belief of broad-spectrum antibiotics are more effective in preventing SSIs, low adherence to healthcare professionals to hospital protocols. It might be also due to the unavailability of culture and sensitivity test which obliges health practitioners to use a broad spectrum of antimicrobials to cover the most likely pathogen.

However, the finding of our study is not in line with the study conducted in South Africa, and Botswana where cefazolin and cefotaxime were the most frequently utilized antibiotics^24,25^. This difference might be due to variations in drug availability, or the types of procedures performed for the patients.

Appropriate use of SAP decreases the occurrence of SSI while inappropriate use of antimicrobial agents such as inappropriate selection, timing, and duration are associated with an increase in the prevalence of antibiotic resistance, cause adverse drug reactions, and increased risk of surgical site infections^15,21^. The present study revealed that only about 22.1% of surgical antibiotic prophylaxis used was appropriate as per available guidelines^6,7^. This was in line with a study conducted at Ayder Hospital which reported 21.9% compliance^23^. The finding was lower than a study conducted in France where 51% of patients received SAP according to guidelines^26^ but higher than the study conducted at Borumeda Hospital^17^. This variation might be due to variations in the experience of professionals or variations in drugs available at a particular hospital.

In our study, inappropriate antibiotic selection was the dominant (62.2%) reason for inappropriate antibiotic use for surgical prophylaxis. This supports the finding of similar studies conducted at Nekemte Referral Hospital.5%)^8^, Ayder (north Ethiopia) (89.5%)^23^, and Palestine (81.5%)^27^. The finding was not concordant with the finding of a study conducted in Australia^5^ where the most common reason for inappropriate procedural SAP was incorrect timing (44.9%), and it was lower than the finding of a study at the Gaffrée e Guinle University Hospital where the choice of the administered antibiotic was considered correct in 97.9% of patients^28^. The difference might be due to in availability of first-line medication and poor implementation of guidelines.

Many guidelines recommend single-dose surgical prophylaxis and the duration should not be more than 24 h^6–8^. In our study, the duration of SAP administration was not concordant in 28 (26.8%) of patients. This was consistence with a study conducted at Hawassa University Referral Hospital where the duration of SAP administration is concordant in 18.4% of patients taking antibiotics^22^. However, it was lower than the finding of a study conducted in Palestine where only 31.8% of patients received an antibiotic for an appropriate duration^27^. This difference could be due to differences in the knowledge of health professionals across different healthcare settings.

Another common failure of guidelines compliance investigated in this study was the wrong timing of antibiotics. In the present study, 24.2% of patients were exposed to the wrong timing of antibiotics administration. This supports the finding of the study conducted in Dessie, Palestine, and Jimma where (59.8%, 48.6%, and 56%) of patients received their first dose at the appropriate time respectively^15,27,29^.

In our study, the duration of surgery longer than an hour and inappropriate use of SAP was identified as independent predictors of surgical site infection occurrence. Similar to our study, studies conducted in different parts of Ethiopia reported that a prolonged duration of surgery (more than an hour) was associated with increased risks of SSI^1,15,20,30^. Another study conducted in Hawassa also revealed that the inappropriate use of poor surgical antimicrobial prophylaxis increases the development of antimicrobial resistance, and SSI^22^.

Many other studies reported the association of SSI with, comorbidity, cigarette smoking, alcohol use, older age, contaminated and dirty wound class, failure to receive antibiotic prophylaxis or delayed initiation of antibiotic prophylaxis, prolonged preoperative hospital stay, previous hospitalization or surgery, and emergency surgical cases^13–17^.

## Conclusion

The higher rate of SSI in the study area despite the institution of surgical antibiotic prophylaxis calls for the consideration of definitive laboratory diagnosis with culture and sensitivity study to come up with recommended antibiotics usage. Bacterial culture confirmation should be done for all patients who developed clinical signs and symptoms of surgical site infections. Policymakers should pinpoint the establishment and implementation of an antimicrobial and diagnostic stewardship approach that will produce better patient outcomes in the future. Health providers who are engaged in surgical ward service should follow the recommendation of guidelines, to come up with the appropriate use of prophylaxis antibiotics in terms of selection, time of administration, and duration of prophylaxis. Including clinical pharmacists in the surgical ward service team should be also considered to ensure proper surgical antibiotic prophylaxis utilization. Moreover, appropriate prophylactic drugs like cefazolin should be procured and availed to prescribers.

### Limitations of the study

The present study period was short which resulted in a small sample size. Variables related to health professionals, antiseptics used for patient preparation, methods used for equipment sterilization, and type of anesthesia used were not included due to resource shortage.

## Data Availability

The authors confirm that data used to support the findings of this study are available from the corresponding author upon request.
